# Large cardiac wall hematoma with rapid growth: a case report of a potentially catastrophic complication during cardiac surgery

**DOI:** 10.1186/s13019-021-01478-y

**Published:** 2021-04-15

**Authors:** Kimihiro Kobayashi, Tetsuro Uchida, Yoshinori Kuroda, Atsushi Yamashita, Eiichi Ohba, Shingo Nakai, Tomonori Ochiai

**Affiliations:** Second Department of Surgery, Faculty of Medicine, Yamagata University, 2-2-2 Iida-Nishi, Yamagata, 990-9585 Japan

**Keywords:** Cardiac wall hematoma, Complication, Cardiac surgery

## Abstract

**Background:**

Rapid growth of cardiac wall hematoma is a rare but potentially fatal complication of cardiac surgery. However, its pathophysiology and optimal management remain undefined.

**Case presentation:**

Here we present a rare case of a large cardiac wall hematoma in the right ventricle during a thoracic aortic and valvular surgery. The hematoma expanded rapidly with epicardial rupture during cardiopulmonary bypass. We could establish non-surgical hemostasis and prevent further expansion of hematoma by early weaning of the cardiopulmonary bypass, followed by the administration of protamine and manual compression by hemostatic agent application. His postoperative recovery was uneventful and upon computed tomography analysis, the hematoma was observed to have absorbed completely at 1 week postoperatively. The patient is doing well 1 year after the surgery without evidence of recurrent cardiac wall hematoma on follow-up computed tomography.

**Conclusions:**

Cardiovascular surgeons should bear in mind this potentially catastrophic complication during cardiac surgery. Because of the vulnerability of the cardiac wall at the area of the hematoma, we believe that a hemostatic approach without sutures may be effective for this lethal complication.

## Background

Small cardiac wall hematoma (CWH) is often observed during cardiac surgery under cardiopulmonary bypass (CPB) which requires heparin administration. Although rapid intraoperative growth of a hematoma to a considerable size is rare, it might result in catastrophic complications associated by hemodynamic deterioration [1–3], and its mortality rate is as high as 23% [1]. Although the precise mechanism of this fatal condition is unclear, some pathophysiologies, such as intramyocardial dissecting hematoma and subepicardial hematoma, are postulated. Large CWH has been reported as a complication of myocardial infarction, percutaneous coronary intervention, ablation of ventricular tachycardia, and chest trauma; however, it has rarely been reported as a complication of cardiac surgery [1]. We describe a case of a rapid growing right ventricular CWH with epicardial rupture during simultaneous thoracic aortic and cardiac surgery. Written informed consent was obtained from the patient for the publication of this article.

## Case presentation

**Fig. 1:**
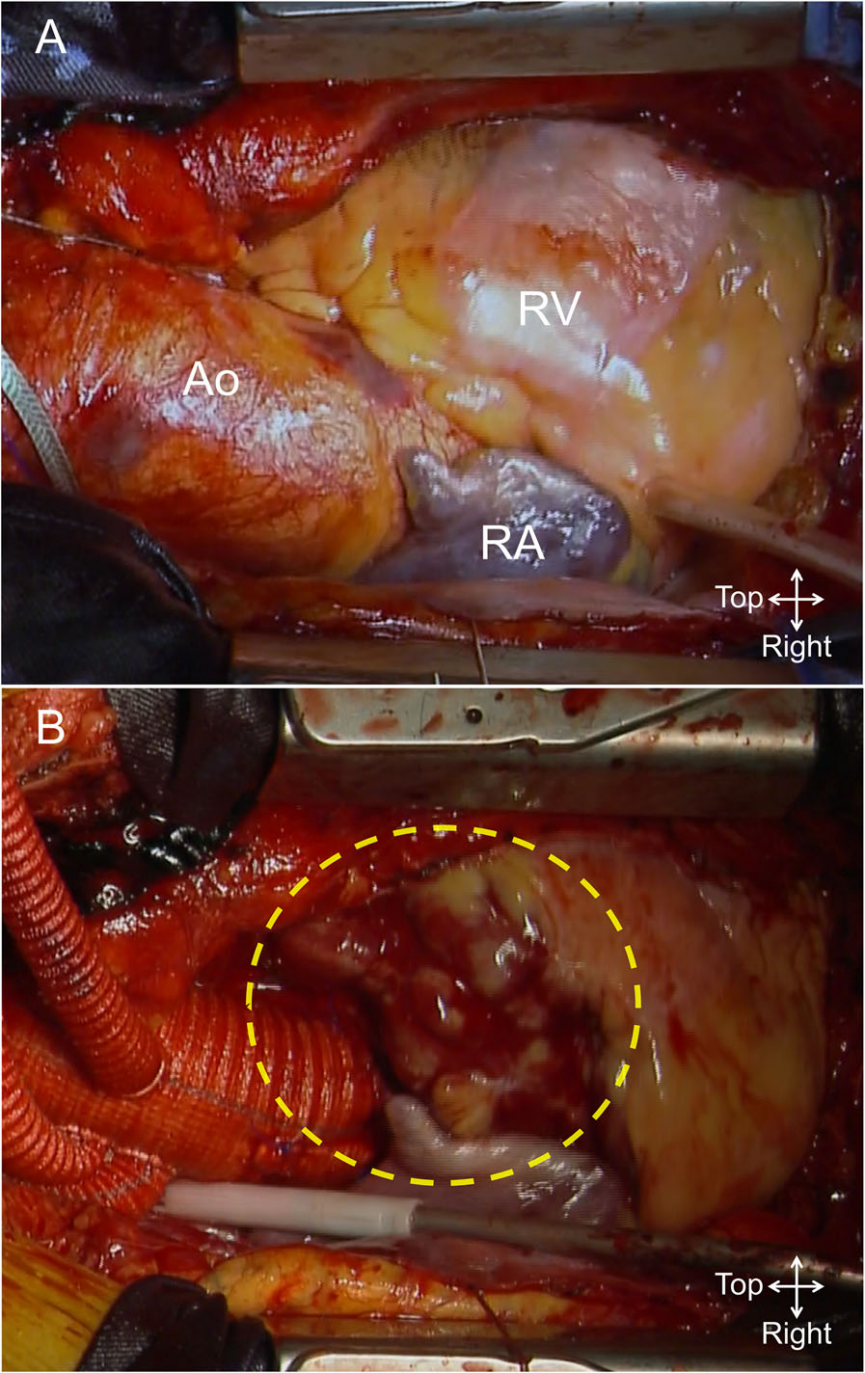
Intraoperative images. **a** The heart before occurrence of cardiac wall hematoma. **b** Cardiac wall hematoma at the right ventricle (dotted circle). Ao, aorta; RA, right atrium; RV, right venticle

A 79-year-old, hypertensive, diabetic, male patient with a thoracic aortic aneurysm and aortic valve stenosis was scheduled for total aortic arch replacement and simultaneous aortic valve replacement. Following sternal entry, CPB was established by right axillary and femoral arterial cannulation with bicaval cannulation. After aortic cross-clamping, myocardial protection was achieved with antegrade, retrograde, and selective cold blood cardioplegia without any technical problem. The aortic valve was replaced with a 21-mm bioprosthesis (Inspiris Resilia, Edwards Life Sciences, Irvine, CA). Subsequent total aortic arch replacement was performed under antegrade selective cerebral perfusion with moderate hypothermic circulatory arrest. After aortic declamping, a small point of oozing hemorrhage was identified on the epicardial surface of the right ventricular outflow tract. The oozing was controlled by hemostatic agent application, followed by the reconstruction of the arch vessels. The surgical maneuver was completed without any technical difficulty; however, we noticed the progressively expanding hematoma at the right ventricular surface to the size of a Japanese rice ball measuring 50 × 50 mm (Fig. 1). The hematoma was hard, and the epicardial surface was dark red and notably swollen with multiple oozing ruptures. There was no evidence of ST-T changes on electrocardiogram. No cardiac wall motion abnormalities were observed on transesophageal echocardiography. Because of the rapid growth of the hematoma, we decided that early discontinuation of CPB and protamine administration should be mandatory. After termination of CPB, followed by the administration of protamine and fresh frozen plasma/platelet transfusion, manual compression with hemostatic agents such as Surgicel (Ethicon, Somerville, NJ, USA) and Tachosil (CSL Behring, Tokyo, Japan) were applied for 15 min. Thereafter, hemostasis was achieved on the epicardial surface, and the hematoma did not expand further without hemodynamic deterioration. The patient showed uneventful postoperative course. Postoperative computed tomography showed hematoma absorption, and transthoracic echocardiography revealed normal ventricular function of both the right and left ventricles. He is doing well 1 year after surgery.

## Discussion and conclusions

When a small hematoma spreads along the myocardial fibers or epicardial tissues, CWH could form. Furthermore, the expanding hematoma could tear the surrounding microvessels, causing further expansion of the hematoma to a considerable size [2]. Because it is difficult to elucidate the precise mechanism of CWH formation intraoperatively, CWH is sometimes considered as an intramyocardial dissecting hematoma or a subepicardial hematoma. According to the literature, CWH has been reported as a complication of acute myocardial infarction, and the mechanism is deemed to be ischemia-induced endothelial cell damage [1]. In percutaneous coronary intervention, direct coronary artery injury and disruption of the interstitial and basal membrane from activated metalloproteinases during reperfusion is speculated to be the cause of CWH [1]. Conversely, cardiac surgery-related CWH is a rare complication; however, it has been reported in mitral valve surgery, coronary artery bypass grafting, and ventricular septal defect repair [1–3]. Because of its rarity, its exact mechanism still remains unclear.

To the best of our knowledge, there have been no reports of CWH associated with thoracic aortic and aortic valvular surgery, as in our case. The possible mechanisms of CWH formation in our present case might include microvessel injury in the cardiac wall induced by (1) excessive traction of the tissue surrounding the right ventricular outflow tract (especially during aortic valve replacement or proximal aortic anastomosis); (2) mechanical damage to the heart externally due to forceps, suction tubes, etc.; (3) mechanical damage to the heart internally due to the Swan-Ganz catheter; and (4) inappropriate cardioplegia perfusion such as excessive perfusion pressure, inadequate myocardial protection, reperfusion injury, and a no-reflow phenomenon [2, 4]. Considering these mechanisms, it is not clear in any case; however, the surgeon should bear in mind the careful and gentle manipulation throughout the surgical maneuver.

The appropriate treatment of CWH during surgery remains controversial. Conservative treatments for hemodynamically stable cases have been described with favorable outcomes in recent years [2, 5]. However, CWH can sometimes be more lethal owing to adverse hemodynamic effects of the hematoma (e.g., ventricular outflow tract stenosis and coronary artery compression), arrhythmias, and bleeding from epicardial rupture, in which case surgical treatment, including removal of the hematoma and hemostatic procedures, should be performed urgently [2, 6]. In fact, surgical removal of CWH is difficult in most cases, and conservative treatment, such as compression hemostasis, early termination of CPB, and blood transfusion, is therefore inevitable. A successful non-surgical hemostatic procedure has been reported [3], and we believe that this procedure is reasonable for use in hemostasis because the site of CWH and the surrounding tissue may be fragile owing to inflammation.

Despite very large hematoma in our present case, radical hematoma removal was not required because there was no hemodynamic deterioration. Fortunately, we successfully controlled hematoma expansion and achieved hemostasis by non-surgical repair using manual compression with hemostatic agents combined with early weaning of CPB and transfusion. In such cases, the hematoma status should be monitored postoperatively by echocardiography, computed tomography, or cardiac magnetic resonance [1, 2, 5] because CWH may grow after surgery, especially in patients receiving anticoagulation [6].

In conclusion, cardiovascular surgeons should be fully aware that CWH can occur during cardiac surgery. It is important that we deal with this lethal complication at the earliest if a CWH is suspected or observed during cardiac surgery. Additionally, a combination of the immediate termination of CPB and non-surgical hemostatic procedures may be effective for this complication.

## Data Availability

The data are not available for public access due to patient privacy concerns but are available from the corresponding author upon reasonable request.

## Abbreviations

CPB: Cardiopulmonary bypass
CWH: Cardiac wall hematoma

## References

[1] Leitman M, Tyomkin V, Sternik L, Copel L, Goitein O, Vered Z (2018). Intramyocardial dissecting hematoma: two case reports and a meta-analysis of the literature. Echocardiography..

[2] McGrath T, Ushukumari D, Canale L, Gillinov M (2015). Dissecting intramyocardial hematoma after robotic mitral valve repair. Ann Thorac Surg.

[3] Ariyama J, Imanishi H, Nakagawa H, Kitamura A, Hayashida M (2010). Epicardial hematoma and myocardial ischemia following application of starfish stabilizer: an uncommon complication of the device. J Anesth.

[4] Vargas-Barrón J, Roldán FJ, Romero-Cárdenas Á, Vázquez-Antona CA (2013). Intramyocardial dissecting hematoma and postinfarction cardiac rupture. Echocardiography..

[5] Pontone G, Bertella E, Andreini D, Pepi M, Polvani G (2014). Postoperative dissecting ventricular haematoma: a conservative strategy with a cardiac magnetic resonance imaging follow-up. Eur Heart J Cardiovasc Imaging.

[6] Mart CR, Kaza AK (2011). Postoperative dissecting ventricular septal hematoma: recognition and treatment. ISRN Pediatr.

